# Intraosseous therapy: harnessing the potential of tissue regeneration and repair

**DOI:** 10.3389/fmed.2026.1775823

**Published:** 2026-05-08

**Authors:** Mikhail Yu. Artamonov, Inessa A. Minenko

**Affiliations:** 1 Department of Physical Medicine and Rehabilitation, Penn Medicine Princeton Health, Plainsboro, NJ, United States; 2 Department of Sports Medicine and Medical Rehabilitation, Sechenov First Moscow State Medical University, Moscow, Russia

**Keywords:** biomaterials, bone fracture repair, bone regeneration, growth factors, intraosseous therapy, spinal fusion, stem cells, tissue regeneration

## Abstract

Tissue regeneration and repair remain significant challenges in various medical fields, particularly in the context of musculoskeletal conditions. Intraosseous therapy has emerged as a promising approach, harnessing the body’s intrinsic regenerative potential to promote bone and tissue regeneration. This review provides a comprehensive overview of intraosseous therapy, including its principles, strategies, and clinical applications. The bone biology and regeneration processes are discussed, along with the roles of cellular components such as osteoblasts, osteoclasts, osteocytes, and mesenchymal stem cells. Intraosseous therapy encompasses various approaches, including Bone marrow aspirate concentrate (BMAC), platelet-rich plasma (PRP), stem cell-based therapies (mesenchymal stem cells, induced pluripotent stem cells, and other sources), biomaterials and scaffolds, and growth factors. The clinical applications of intraosseous therapy span bone fracture repair and non-union, spinal fusion, craniofacial and maxillofacial reconstruction, osteonecrosis and avascular necrosis, and osteoporosis and bone defects. While intraosseous therapy holds significant promise, challenges related to regulatory and ethical considerations, standardization and quality control, clinical translation and commercial viability, and future research directions in personalized medicine, advanced biomaterials, combination therapies, and *in vivo* monitoring must be addressed. Ultimately, intraosseous therapy represents a rapidly evolving field with the potential to revolutionize tissue regeneration and repair, offering improved outcomes and enhanced quality of life for patients suffering from various musculoskeletal conditions.

## 1 Introduction

Tissue regeneration and repair are an intriguing area of study in biomedical research, with the potential to significantly transform the treatment of many illnesses and injuries. One of the promising areas in this research is intraosseous treatment, a novel method that utilizes the body’s natural ability to regenerate and mend bones. This innovative approach has attracted considerable interest because of its capacity to tackle many bone-related disorders and increase healing results (1). Bone tissue has a crucial function in the human body, serving as a framework, safeguarding essential organs, regulating mineral balance, and allowing movement (2). Nevertheless, bone abnormalities, including fractures, non-unions, osteoporosis, and degenerative illnesses, may have a considerable influence on an individual’s quality of life and place a large cost burden on healthcare systems (3). Historically, the management of these disorders has mainly depended on invasive surgical procedures, which may be linked to potential dangers, problems, extended recuperation times, and significant healthcare expenses (4). Intraosseous treatment is a revolutionary approach to bone regeneration and repair that harnesses the body’s own healing processes and delivers therapeutic substances directly into the bone marrow cavity. This technique seeks to enhance and amplify the natural ability of bone tissue to regenerate (5).

The bone marrow, located in the medullary cavity of bones, contains mesenchymal stem cells (MSCs) and other progenitor cells that may develop into several kinds of cells, including osteoblasts, which play a vital role in bone creation (6). Intraosseous treatment is based on the understanding that the bone marrow microenvironment is crucial in controlling stem cell activity and directing the process of bone regeneration (1). The microenvironment, which consists of numerous biological components, extracellular matrix proteins, and signaling molecules, plays a crucial role in determining the fate of stem cells and their capacity to transform into bone-forming cells (7). Researchers aim to improve recruitment, proliferation, and differentiation of stem cells by introducing therapeutic molecules directly into the microenvironment. This approach ultimately promotes more efficient and quicker bone repair and regeneration (8).

An important benefit of intraosseous treatment is its minimally invasive nature, which often entails injecting medicinal drugs into the bone marrow cavity by a tiny incision or percutaneous access (4). This method aims to limit the physical damage caused by surgery and perhaps decrease the likelihood of consequences that are commonly linked with more invasive operations, such as infection, nerve injury, and significant blood loss (9). Intraosseous therapy has the ability to provide tailored and targeted therapies by utilizing patient-specific stem cells or customizing therapeutic molecules based on individual requirements and genetic profiles (10). Intraosseous treatment involves several tactics and procedures, such as the utilization of bone marrow aspirate concentrate (BMAC), platelet-rich plasma (PRP), and stem cell-based therapies (11). BMAC is a highly concentrated solution of bone marrow aspirate that includes a plentiful supply of mesenchymal stem cells (MSCs), hematopoietic stem cells, and growth factors. It may be administered directly into the bone marrow cavity to facilitate the healing process (12).

Platelet-rich plasma (PRP) is a concentrated solution of platelets and growth factors obtained from the patient’s own blood. It is used to improve the healing and regrowth of tissues (13). Stem cell-based treatments have become a hopeful method in intraosseous therapy, utilizing the regeneration capabilities of several stem cell sources (14). MSCs obtained from bone marrow, adipose tissue, or other origins, have shown significant capability in stimulating bone regeneration by their capacity to transform into osteoblasts and produce various growth factors and cytokines that facilitate bone healing (15). Moreover, iPSCs which are secreted from somatic cells using reprogramming techniques, provide a flexible means of producing patient-specific stem cells for bone regeneration. This method avoids the ethical issues linked to embryonic stem cells (16). In addition, the combination of biomaterials and scaffolds has directed toward more efficient opportunities to improve the efficacy of intraosseous treatment (17).

Biomaterials, derived from either natural sources (such as collagen and hydroxyapatite) or synthetic materials (such as polymers and ceramics), serve multiple functions including providing structural support, promoting cell attachment and growth, and allowing for controlled release of therapeutic substances (18). In addition, the combination of natural and synthetic components in composite biomaterials provides customized qualities to fulfill specific regeneration requirements (19). Growth factors and signaling molecules are essential in the bone regeneration process since they control cellular processes, including proliferation, differentiation, and matrix synthesis (20). Bone morphogenetic proteins (BMPs) are growth factors that belong to the transforming growth factor-beta (TGF-β) class. They have been widely researched because of their potential to induce bone formation and promote the transformation of MSCs into osteoblasts. Vascular endothelial growth factor (VEGF) is a significant signaling molecule that stimulates angiogenesis, a crucial process for the repair and regeneration of bones (21).

Additional growth factors and signaling molecules, such as fibroblast growth factors (FGFs), insulin-like growth factors (IGFs), and Wnt signaling proteins, have also been investigated for their potential to improve bone regeneration (22). Although intraosseous therapy shows promise, there are still several challenges that need to be addressed. These challenges include optimizing the methods of delivery, ensuring long-term safety and effectiveness, dealing with regulatory and ethical issues, and achieving successful application in clinical settings and commercial viability (23). Current research is primarily focused on comprehending the fundamental mechanisms, creating sophisticated biomaterials and delivery systems, investigating combination therapies, and implementing techniques for monitoring and tracking in living organisms to optimize the therapeutic advantages (20).

As a result, the purpose of this article is to provide a narrative assessment of existing biological, preclinical, and clinical findings to contextualize the evolving function of intraosseous therapy in tissue and repair regeneration.

## 2 Review methodology

This article was written as a narrative review with the goal of presenting a comprehensive and concept-driven overview of intraosseous treatment and its function in tissue regeneration and repair. A thorough literature search was conducted utilizing the internet databases PubMed/MEDLINE, Scopus, and Google Scholar to find relevant peer-reviewed papers published between January 2000 and March 2024. The following keywords were included in the search: “intraosseous therapy,” “bone regeneration,” “bone marrow aspirate concentrate,” “platelet-rich plasma,” “mesenchymal stem cells,” “biomaterials,” “scaffolds,” “growth factors,” “bone repair,” and “spinal fusion.” Reference lists for selected papers were manually searched to find more relevant publications.

Moreover, original research articles (*in vitro*, animal, and clinical investigations), randomized controlled trials, observational studies, and only relevant English-language systematic reviews were all considered eligible. Editorials, conference abstracts that lacked complete data, and research unrelated to musculoskeletal or bone regeneration were removed. The selection of studies was based on their relevance to the intraosseous therapy conceptual framework, which includes biological mechanisms, therapeutic techniques, biomaterials, growth factors, and clinical applications. Data extraction was centered on study design, therapeutic strategy, biological or clinical outcomes, and stated benefits or limits. Given the narrative aspect of this research, findings were synthesized qualitatively rather than via formal meta-analysis.

## 3 Bone biology and regeneration

### 3.1 Structure and composition of bone tissue

**FIGURE 1:**
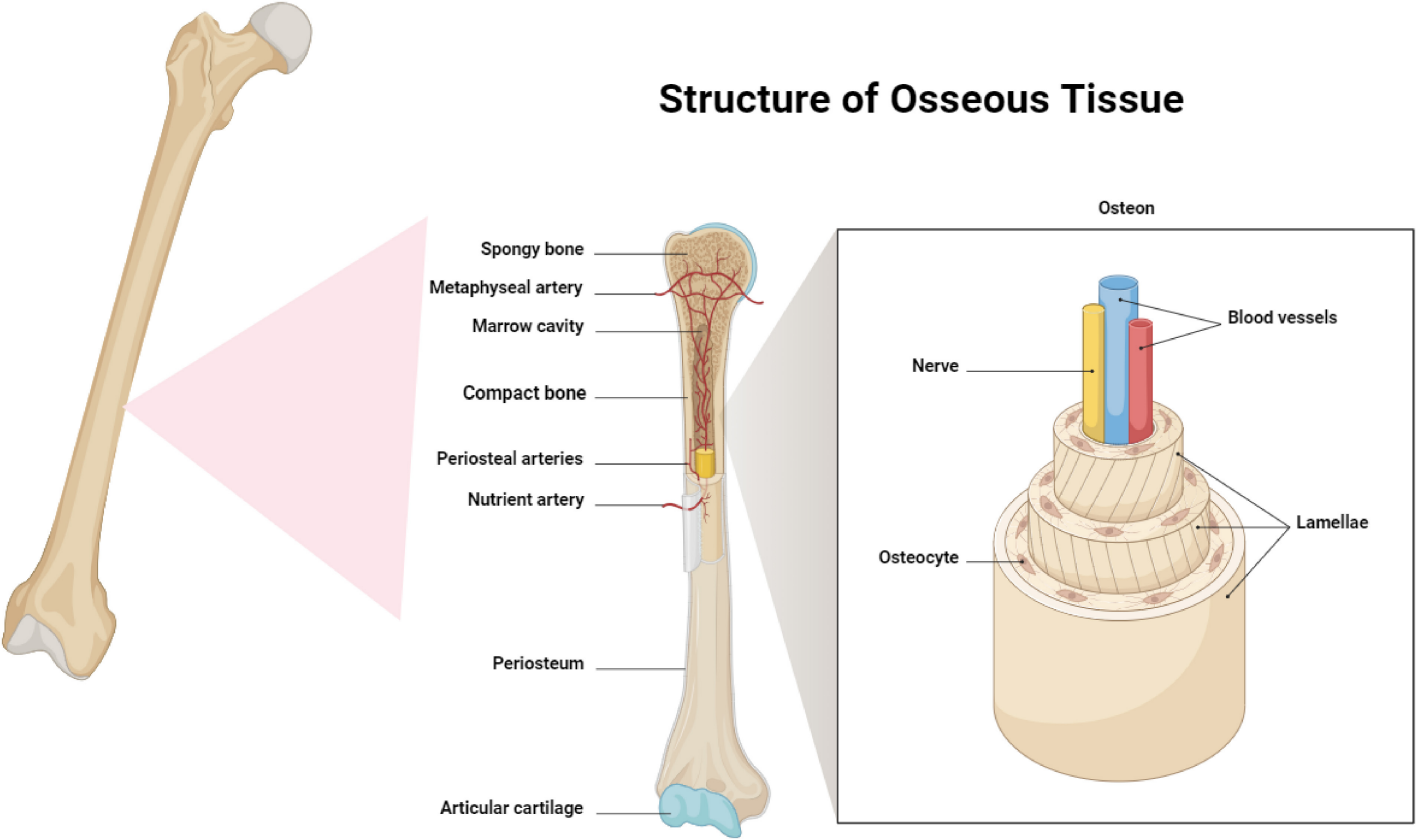
Structure of osseous tissues, this diagram illustrates the intricate structure of osseous tissue, highlighting the compact and spongy bone composition, vascularization through periosteal and nutrient arteries, and the detailed microstructure of an osteon with its lamellae, blood vessels, and innervation.

Bone tissue is a specialized kind of connective tissue that plays vital roles in the human body, including providing structural support, permitting mobility, and maintaining mineral balance (2). The composition of bone consists of two primary types (Figure 1): cortical (compact) bone and cancellous (trabecular) bone. Cortical bone, also known as compact bone, is the highly dense and rigid outer layer that comprises the shaft of long bones and the outer covering of flat bones (24). The bone is structured in concentric cylindrical units known as osteons or Haversian systems. These units are composed of concentric layers of mineralized matrix that around a central canal, known as the Haversian canal, which houses blood vessels and nerves. The exceptional strength and load-bearing capabilities of cortical bone are attributed to its highly ordered structure. Cancellous bone, also known as trabecular bone, is the inner layer of bone that is porous and spongy in nature. It is mostly located in the ends of long bones and the core of flat bones (25). The structure consists of a network of interconnected trabecula, which are tiny rods or plates, forming a honeycomb-like pattern (26). This marrow is vital for hematopoiesis, the formation of blood cells, and also acts as a storage site for mesenchymal stem cells that are necessary for the process of bone regeneration (6). Both cortical and cancellous bone consist of an organic matrix, largely consisting of type I collagen fibers, and an inorganic mineral phase, made of hydroxyapatite crystals (27). The organic matrix imparts flexibility and toughness to bone tissue, whereas the inorganic mineral phase enhances its hardness and compressive strength. The unique arrangement of components gives bone its exceptional mechanical characteristics, allowing it to endure different forces and pressures while retaining a certain level of flexibility.

### 3.2 Cellular components and their roles

**FIGURE 2:**
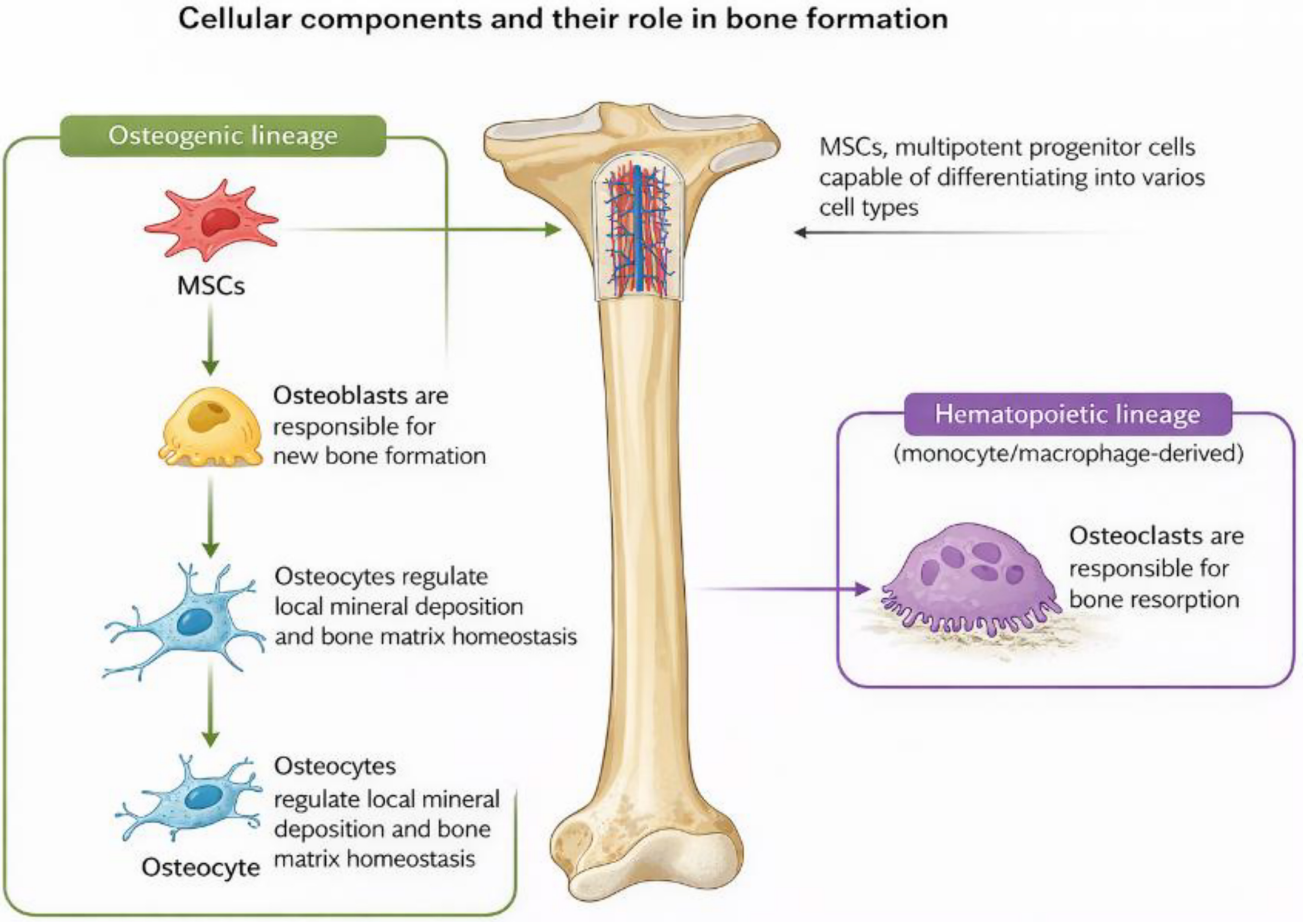
Cellular components and their role in bone formation, figure captures the cellular hierarchy within bone physiology, showing osteoclasts that resorb aged bone, osteoblasts which construct new bone matrix, and osteocytes that orchestrate the mineral balance. Central to the process are mesenchymal stem cells, depicted as the source for these critical bone-forming cells.

Bone tissue is a dynamic and highly organized structure composed of various cellular components that play crucial roles in its formation, remodeling, and maintenance. The four primary cell types involved in bone biology are osteoblasts, osteoclasts, osteocytes, and mesenchymal stem cells (MSCs) indicated in Figure 2.

#### 3.2.1 Mesenchymal stem cells (MSCs)

Mesenchymal stem cells (MSCs) are a type of versatile precursor cells that are present in several tissues, such as bone marrow, adipose tissue, and tooth pulp (28). MSCs have the capacity to undergo self-renewal and develop into several types of cells, such as osteoblasts, chondrocytes, and adipocytes (29). Within the field of bone biology, MSCs play a vital role in the synthesis and restoration of bones. They have the ability to transform into osteoblasts and aid in the healing of bone deformities or fractures (30). In addition, mesenchymal stem cells (MSCs) release a variety of growth factors and cytokines that promote the formation of new blood vessels (angiogenesis) and regulate the bone microenvironment (31). The coordinated interplay between these cellular components is essential for maintaining the integrity and function of bone tissue, ensuring a dynamic balance between bone formation and resorption processes.

#### 3.2.2 Osteoblasts

Osteoblasts are crucial for bone formation. They originate from mesenchymal stem cells (32). Their responsibility is in the synthesis and secretion of the organic matrix, which is predominantly, made up of type I collagen. They also play a role in regulating the mineralization of the matrix (33). Osteoblasts synthesize a variety of proteins, including osteocalcin and osteopontin, that play a crucial role in the process of bone production and mineralization (34). In addition, they produce alkaline phosphatase, an essential enzyme for the onset of mineralization (35).

#### 3.2.3 Osteoclasts

Osteoclasts are multinucleated cells that originate from hematopoietic stem cells in the monocyte/macrophage lineage (36). They have the responsibility of carrying out bone resorption, which is a crucial process for bone remodeling and maintaining mineral balance in the body (37). Osteoclasts adhere to the surface of the bone and establish a tightly enclosed space called the resorption lacuna. Within this lacuna, the osteoclasts release enzymes and acids that breakdown the mineralized matrix of the bone (38). The regulation of this process is governed by many signaling pathways, including as the RANK/RANKL/OPG system, which oversees the development and activity of osteoclasts (39).

#### 3.2.4 Osteocytes

Osteocytes are the predominant cells seen in fully developed bone tissue. They originate from osteoblasts and get lodged inside the mineralized matrix throughout the process of bone formation (40). Osteocytes are located within lacunae and to are related each other by their cytoplasmic processes, which extend through canaliculi in the bone matrix (41). Osteocytes play a vital role in detecting and reacting to mechanical stimuli, controlling bone remodeling, and maintaining mineral balance (42). They release a variety of signaling molecules, including as sclerostin and fibroblast growth factors (FGFs), that affect the process of bone production and resorption (40).

### 3.3 Bone remodeling process

**FIGURE 3:**
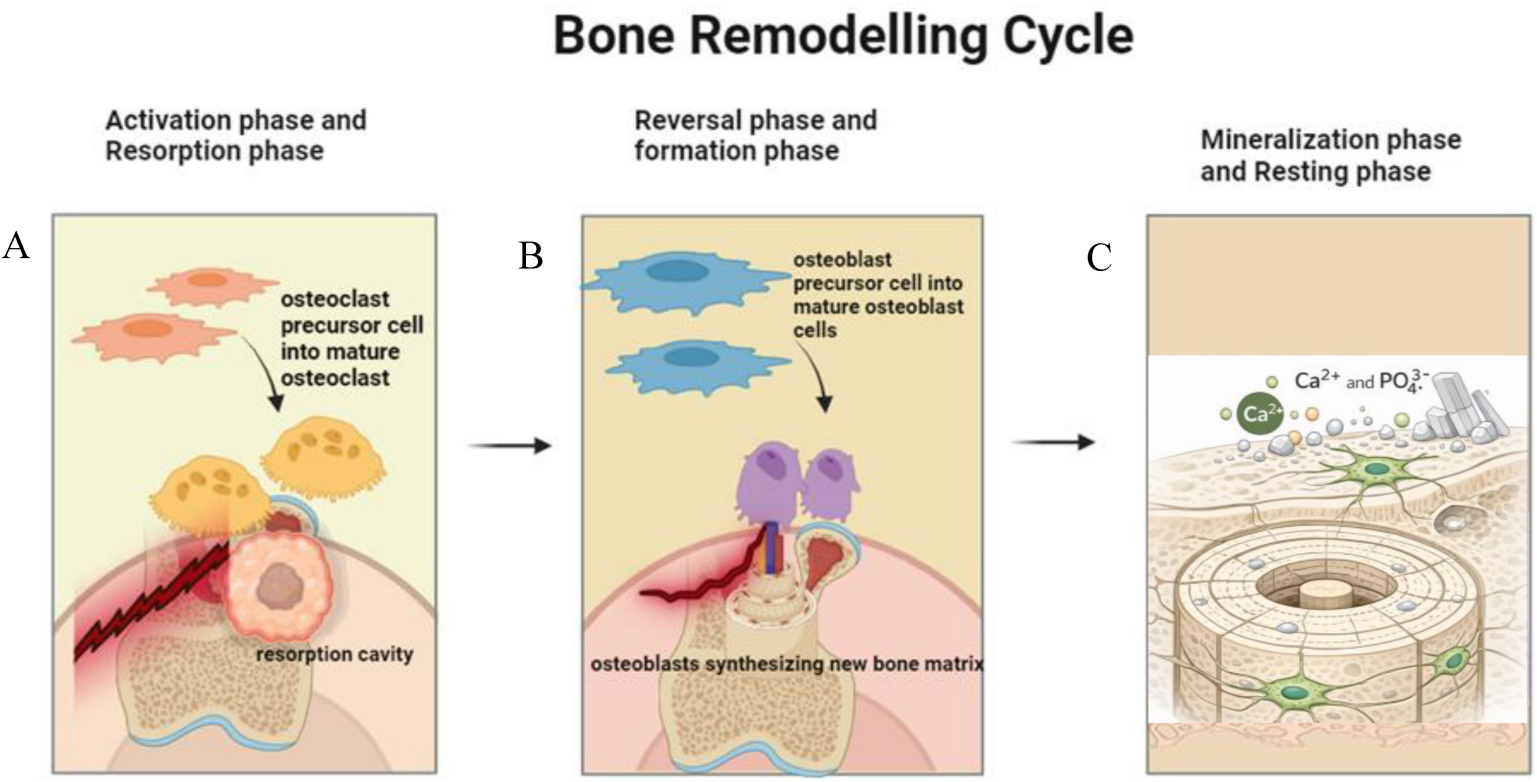
Bone remodeling cycle. **(A)** Activation and resorption stage: Osteoclast precursors are transformed into mature osteoclast, which breaks down bone matrix and forms a resorption cavity. **(B)** Reversal phase and formation stage: At this stage Osteoblast precursors become mature osteoblasts producing new bone mass at the site of resorption. **(C)** Mineralization and resting stage: Differentiating bone is then mineralized (Ca^2+^), resulting in the maturation of a matrix and bone structure is restored in the resting stage.

Bone remodeling is a highly regulated and dynamic process that involves the continuous regeneration and replacement of bone tissue during a person’s lifespan. The function of this mechanism is essential for maintaining the structural integrity and mechanical efficiency of the skeletal system, as well as for regulating calcium balance (43). The bone remodeling process has two distinct stages: bone resorption and bone formation (Figure 3). Osteoclasts and osteoblasts are specialized cells responsible for carrying out these stages, as described by Hadjidakis and Androulakis (44). The bone remodeling cycle starts with the activation phase, during which progenitor cells are recruited to the remodeling site (45). The initiation of this phase is triggered by a variety of physiological and mechanical signals, such as hormones, cytokines, and mechanical stress (46). After being activated, the precursor cells continue a process of differentiation and change into fully mature osteoclasts, which subsequently begin the process of resorption. During the resorption phase, osteoclasts attach to the bone surface and create a tightly contained microenvironment known as the resorption lacuna. Osteoclasts secrete hydrochloric acid and proteolytic enzymes, namely cathepsin K, into this space. These chemicals disintegrate the hardened bone structure and decompose the organic components (37). The formation of a fringed boundary, known as a specialized membrane domain, aids in this process by expanding the surface area for the release and uptake of resorption products (47). Following the resorption phase, there is a second reversal phase when the bone surface is readied for the generation of new bone (48). This step involves the recruitment of mononuclear cells, which remove the resorption lacuna and release signaling molecules that attract and promote osteoblast precursor cells (49). The formation phase starts with the differentiation and migration of precursor cells of osteoblasts to the region where remodeling takes place (50). Mature osteoblasts produce and release the main organic elements of the bone matrix, mainly type I collagen, along with non-collagenous proteins including osteocalcin and osteopontin (2). Afterward, the matrix proceeds through mineralization, which is made possible by the presence of alkaline phosphatase and the formation of hydroxyapatite crystals (35). During the synthesis phase, certain osteoblasts get enclosed inside the recently synthesized bone matrix and undergo differentiation to become osteocytes (51). Osteocytes have a vital function in controlling the process of bone remodeling by detecting mechanical stimuli and releasing signaling molecules that impact the activity of osteoblasts and osteoclasts (40). The process of bone remodeling is closely controlled by a variety of variables, both systemic and local. These factors include hormones (such as parathyroid hormone and estrogen), cytokines (such as interleukins and tumor necrosis factor), and signaling pathways (such as RANK/RANKL/OPG and Wnt/β-catenin) (52). The equilibrium between bone resorption and production is crucial, since any disparities can result in bone illnesses, such as osteoporosis (excessive resorption) or osteopetrosis (impaired resorption) (53).

### 3.4 Challenges in bone regeneration

One significant obstacle in the process of bone regeneration is to ensure sufficient vascularization and nutrition delivery to the newly forming tissue. Bone lesions that exceed a threshold size, typically about 2–3 cm in humans, have challenges in healing because of inadequate vascularization (54). Research has demonstrated that bone transplants that lack proper blood supply experience considerably reduced rates of bone development, with just 10%–20% of the graft volume undertaking the process of generating new bone (55). The biomechanical environment is essential, as insufficient mechanical stress can result in poor bone regeneration and reduced bone mineral density. A research conducted by Preclinical investigations utilizing controlled animal fracture models, they have shown that the absence of mechanical loading is linked with significantly lower bone growth compared to mechanically loaded areas (56). Experimental estimations show a reduction of around one-half; however, these data come from animal models and should be evaluated cautiously when applied to human fracture healing.

Excessive mechanical strain has been proven in experimental and computational models to inhibit bone healing and promote fibrous tissue development. Specific strain thresholds have been proposed in preclinical research; however, these values vary significantly by model system and anatomical region and should not be taken as set limitations in human bone healing (57). Age-related reduction in osteoblast and mesenchymal stem cell (MSC) activity, together with other systemic variables, might hinder bone repair. Studies in elderly animal models have repeatedly shown a significant 50% age-related loss in osteogenic ability and mesenchymal stem cell activity compared to younger controls. Evidence in human populations is still primarily indirect, derived from observational or surrogate marker research (58). In diabetic animal models, experimental studies show a moderate decline in bone production rates when compared to non-diabetic controls. This effect’s strength varies depending on the model and the severity of the illness, and it is unclear how directly it relates to human fracture healing (59). Both infection and inflammation can greatly impede the process of bone repair. Preclinical and observational studies show that infection at bone regeneration sites significantly reduces new bone production as compared to uninfected settings. However, claimed effect sizes vary greatly depending on the pathogen, host response, and treatment environment.

Prolonged inflammation, characterized by consistently high levels of inflammatory cytokines such as TNF-α and IL-1, might impede the process of osteoblast development and the formation of bone (60). Complicated defect shapes and bigger defect dimensions present considerable obstacles for bone repair. Substantial segmental bone abnormalities are associated with a significantly lower likelihood of spontaneous bridging without extra intervention. Preclinical trails reported healing rates vary significantly among species, anatomical sites and fixation techniques, restricting the determination of universal size thresholds.

The rate of healing diminishes exponentially as the size of the defect increases, as demonstrated by Bodde et al. (61), who found that the healing rate drops by 50% for each additional 1 cm in defect size. Inadequate recruitment and differentiation of stem cells might hinder the process of bone repair. Studies conducted in a laboratory setting have demonstrated that mesenchymal stem cells (MSCs) obtained from older donors have a decreased rate of cell division (up to 50% lower) and a diminished ability to develop into bone cells compared to MSCs obtained from younger donors (62). Osteoporosis and other disorders have been linked to altered MSC activity and decreased ability to generate new bone, which hampers bone regeneration (63).

The quantitative impact sizes addressed in this section are based mostly on preclinical or non-comparative research and should be taken as indicating trends rather than definite estimates applicable to human clinical outcomes.

## 4 Intraosseous therapy

Intraosseous treatment involves the direct administration of biological substances, such as stem cells, growth hormones, and other restorative components, into the bone marrow cavity or a specific region of bone damage (64). This technique seeks to utilize the body’s own healing mechanisms and facilitate the regrowth and restoration of bones by supplying the required cellular and molecular elements directly to the injured or damaged area. BMAC refers to a bone marrow product that undergoes little manipulation and has a concentrated amount of mesenchymal stem cells (MSCs), hematopoietic stem cells, and other progenitor cells. It also contains growth factors and cytokines (65). Research has demonstrated encouraging outcomes in utilizing BMAC (bone marrow aspirate concentrate) for the treatment of different bone deformities and non-unions. An example of this is a randomized controlled experiment conducted by Desai et al. (66). The study showed that patients with atrophic non-unions who were treated with BMAC had considerably greater rates of bone union (88% compared to 62%) and quicker healing periods compared to those who received iliac crest bone transplant. Besides its clinical uses, BMAC has demonstrated encouraging outcomes in preclinical investigations. A research conducted by Miar et al. (67) showed that mice treated with BMAC had improved bone regeneration and higher bone volume development in critical-sized calvarial lesions, as compared to the control group. The presence of mesenchymal progenitors and growth factors in BMAC was identified as the cause of this impact. These components encouraged the process of osteogenic differentiation and angiogenesis. Moreover, BMAC can be integrated with other biomaterials and scaffolds to enhance its distribution and efficacy. In a research conducted by Chen et al. (68), the effectiveness of using BMAC (bone marrow aspirate concentrate) combined with a 3D-printed calcium phosphate scaffold was assessed for the treatment of critical-sized femoral lesions in rats. The combination of BMAC and scaffold demonstrated a substantial enhancement in bone growth, vascularization, and mechanical qualities as compared to the scaffold alone or empty defects. Platelet-rich plasma (PRP) is a blood product derived from the patient’s own blood and has a high concentration of platelets. When activated, these platelets release a range of growth factors and cytokines (69). PRP has been well-researched for its capacity to improve bone regeneration and accelerate the healing of fractures. A meta-analysis conducted by Ghaffarpasand et al. (70) revealed that the utilization of Platelet-Rich Plasma (PRP) led to a substantial enhancement in bone healing and a reduction in healing duration among patients with long bone non-union, as compared to control groups. PRP has demonstrated potential in improving spinal fusion results, in addition to its application in non-unions. A meta-analysis revealed that the utilization of Platelet-Rich Plasma (PRP) in spinal fusion operations led to increased fusion rates, enhanced functional results, and decreased pain scores in comparison to control groups who did not receive PRP (71). Therefore, a comparative summary is given in Table 1 to make it easier to compare various intraosseous therapeutic approaches and to show variations in biological potential, translational maturity, and clinical evidence.

**TABLE 1:** Comparative overview of major intraosseous biological therapies.

Therapy	Cell/factor source	Osteogenic potential	Ease of harvest	Regulatory status	Level of clinical evidence	Key advantages	Key limitations
BMAC	Autologous bone marrow	Moderate–high	Moderate (invasive aspiration)	Minimal manipulation (varies by region)	Case series, RCTs	Autologous, rich in progenitors	Variable cell yield
PRP	Autologous blood platelets	Indirect (growth factor–mediated)	Easy	Widely permitted	RCTs, meta-analyses	Low cost, simple	Protocol heterogeneity
BM-MSCs	Bone marrow MSCs	High (preclinical)	Invasive	Advanced therapy	Preclinical, early clinical	Strong osteogenesis	Expansion required
ADSCs	Adipose-derived MSCs	Moderate–High	Easy	Advanced therapy	Preclinical, limited RCTs	High yield	Variable osteogenicity
iPSC-derived MSCs	Reprogrammed somatic cells	High (experimental)	Complex	Highly regulated	Preclinical	Unlimited supply	Safety, cost

### 4.1 Stem cell-based therapies

Stem cell-based intraosseous therapies include a variety of cellular techniques aiming at promoting bone repair via osteogenic differentiation, paracrine signaling, and immunomodulatory effects. Multiple stem cell sources have been investigated, each with varying biological potential, translational maturity, and regulatory complexity. Among these, mesenchymal stem cells are the most widely studied and clinically advanced option, while iPSCs are primarily experimental. The following sections discuss and compare the major stem cell platforms studied for intraosseous applications.

Mesenchymal stem cells are extensively researched and considered a very promising source of stem cells for bone regeneration. This is because they possess the potential to differentiate into many cell types, possess immunomodulatory capabilities, and can produce a variety of growth factors and cytokines (72). Recent research has investigated the utilization of MSCs in conjunction with biomaterials and scaffolds to improve their administration and effectiveness. An investigation conducted by Chen et al. (73) showcased enhanced bone regeneration in a rat calvarial defect model with the utilization of a 3D-printed gelatin-based hydrogel infused with MSCs and bioactive glass nanoparticles. In addition, scientists have examined methods to improve the ability of MSCs to form bone tissue, such as altering their genes or exposing them to certain substances before use. A research conducted by Lee et al. (74) discovered that mesenchymal stem cells (MSCs) that had an increased expression of the transcription factor RUNX2 shown improved ability to differentiate into bone cells and regenerate bone tissue. This was observed when these cells were transplanted into a critical-sized calvarial deficiency in mice.

### 4.2 Induced pluripotent stem cells (iPSCs)

Induced pluripotent stem cells which are formed by reprogramming somatic cells, are being considered as a possible substitute for embryonic stem cells in the field of bone regeneration (75). A recent investigation conducted by Kato et al. (76) showcased the ability of induced pluripotent stem cell (iPSC)-derived mesenchymal progenitor cells (iMPCs) to promote bone formation in a rat model with defects in the skull. The iMPCs exhibited the capacity to develop into osteoblasts and stimulate bone repair, demonstrating similar effectiveness to bone marrow-derived MSCs. Scientists have also investigated alternative sources of stem cells for the purpose of bone repair, including embryonic stem cells (ESCs), adipose-derived stem cells (ADSCs), and dental stem cells (DPSCs). The work conducted by Xu et al. (77) examined the use of mesenchymal stem cells produced from embryonic stem cells (ESC-MSCs) in conjunction with a collagen-hydroxyapatite scaffold for the purpose of repairing bone defects in a rat model. The scaffolds loaded with ESC-MSCs exhibited superior bone growth and vascularization in comparison to the scaffolds without the cells. ADSCs have also shown promise as an alternative to bone marrow-derived MSCs due to their ease of accessibility and similar multi-lineage differentiation potential (78).

## 5 Biomaterials and scaffolds for intraosseous therapy

### 5.1 Natural biomaterials

Collagen, hydroxyapatite, and chitosan are natural biomaterials that have been intensively studied for their potential in bone regeneration. This is because they are biocompatible, biodegradable, and capable of replicating the natural bone microenvironment (79). Collagen-based scaffolds have demonstrated encouraging outcomes in stimulating osteogenic differentiation and the synthesis of bone. A study conducted by Liu et al. (80) showed that the use of a collagen-hydroxyapatite scaffold combined with mesenchymal stem cells (MSCs) and platelet-rich plasma (PRP) resulted in improved bone regeneration in a rat calvarial lesion model. Hydroxyapatite, a mineral found in bones, is commonly employed as a biomaterial for bone regeneration because of its strong compatibility with living tissue and its ability to promote bone growth (81).

### 5.2 Synthetic biomaterials

Synthetic biomaterials, such as polymers [e.g., polycaprolactone, poly (lactic-co-glycolic acid], ceramics (e.g., tricalcium phosphate, bioactive glasses), and metals (e.g., titanium alloys), provide adjustable characteristics and predictable rates of deterioration for the purpose of bone regeneration (82). Polycaprolactone (PCL) is a commonly employed biodegradable polymer in the field of bone tissue engineering owing to its favorable mechanical characteristics and compatibility with living organisms (83). Scaffolds made from this material are commonly employed in the construction of structures for different tissue engineering purposes, such as the regeneration of bone (84). Studies have demonstrated that bioactive glasses, namely those that include boron, can promote the formation of new bone tissue (osteogenesis) and the growth of new blood vessels (angiogenesis). This is attributed to their ability to quickly convert into hydroxyapatite, as well as the beneficial effects of the dissolved ions they release (85). Mesoporous bioactive glasses, such as MBG-75S, have been discovered to enhance these activities by releasing calcium, phosphorous, and silicon ions (86).

### 5.3 Composite biomaterials

**FIGURE 4:**
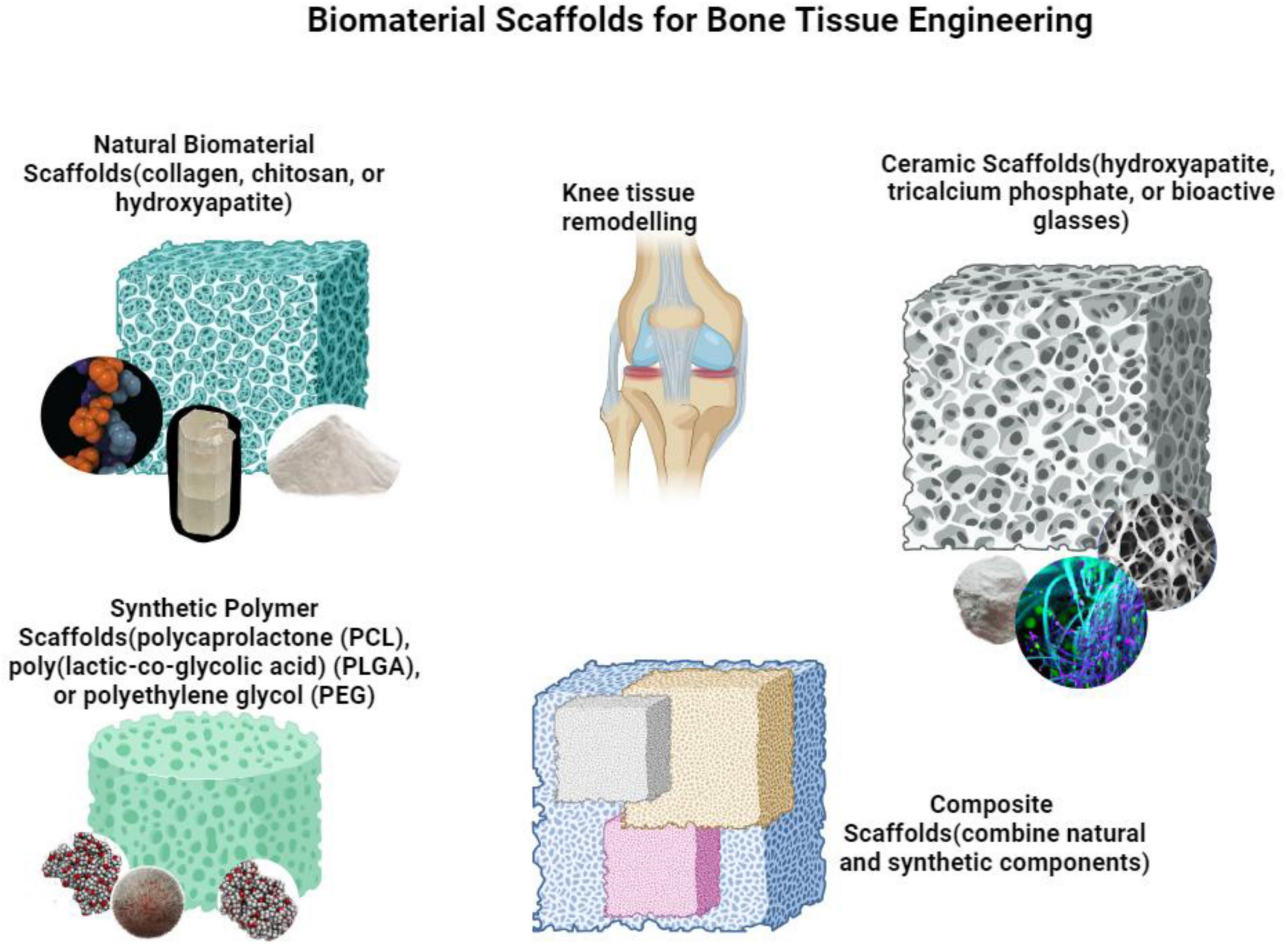
Biomaterial scaffolds for bone tissue regeneration, figure illustrates various scaffolding materials used in bone tissue engineering: natural biomaterials like collagen, synthetic polymers such as polycaprolactone (PCL) and poly(lactic-co-glycolic acid) (PLGA), ceramics including hydroxyapatite, and composite scaffolds that merge natural and synthetic elements to support knee tissue remodeling and bone regeneration.

Composite biomaterials, which incorporate both natural and synthetic elements, have garnered considerable interest in the realm of bone tissue engineering because they may harness the benefits of each individual material (Figure 4). The purpose of these hybrid scaffolds is to replicate the intricate structure and composition of natural bone, creating an environment that promotes the attachment, growth, and specialization of cells involved in bone formation Chatzinikolaidou (87). An effective method includes integrating bioactive ceramics, such as hydroxyapatite or bioactive glasses, with polymeric matrices like polycaprolactone (PCL) or poly(lactic-co-glycolic acid) (PLGA). Karimzadeh Bardeei et al. (88) showed that a 3D-printed PCL/npW composite scaffold accelerated the process of mesenchymal stem cells (MSCs) transforming into bone cells (osteogenic differentiation) and boosted bone regrowth in a rabbit femoral lesion model. The study conducted by Prosecká et al. (89) discovered that the use of a scaffold made of Coll/HA/PCL, along with MSCs and a solution rich in thrombocytes, effectively enhanced the process of bone regeneration in lesions identified in the femoral condyle of rabbits. Table 2 summarizes the essential features, advantages, and limitations of commonly used biomaterials and scaffolds for intraosseous treatment.

**TABLE 2:** Key biomaterials and scaffolds used in intraosseous therapy.

Biomaterial	Type	Mechanical properties	Bioactivity	Degradation	Typical applications	Strengths	Limitations
Collagen	Natural	Low	High	Fast	Craniofacial defects	Biocompatible	Weak mechanics
Hydroxyapatite	Ceramic	High	Osteoconductive	Slow	Load-bearing bone	Bone-mimetic	Brittle
PCL	Synthetic polymer	Moderate–high	Low	Slow	3D-printed scaffolds	Printable	Poor bioactivity
Bioactive glass	Ceramic	Moderate	High	Controlled	Bone regeneration	Ion release	Brittleness
Composite scaffolds	Hybrid	Tunable	High	Tunable	Large defects	Balanced properties	Manufacturing complexity

## 6 Growth factors and signaling molecules

Bone morphogenetic proteins, term for bone morphogenetic proteins, are a collection of growth factors that play crucial roles in regulating bone synthesis, restructuring, and restoration (90). Several bone morphogenetic proteins (BMPs), namely BMP-2 and BMP-7, have been extensively studied and used in strategies targeting the regeneration of bone tissue. In a clinical trial conducted by Govender et al. (91) it was found that the use of recombinant human BMP-2 (rhBMP-2) on an absorbable collagen sponge resulted in significantly increased bone growth and successful healing in patients with open tibial fractures, compared to the use of autograft therapy. In addition to directly promoting bone tissue formation, BMPs have also been shown to enhance the differentiation of mesenchymal stem cells (MSCs) into bone cells and facilitate the development of new blood vessels, which is crucial for bone repair (92). VEGF plays a critical role in regulating angiogenesis and is essential for promoting the growth of blood vessels during bone regeneration (93). Adequate angiogenesis is essential for the delivery of nutrients, oxygen, and progenitor cells to the regeneration site. Kempen et al. (94) did aresearch that demonstrated the positive effects of administering VEGF using a polymeric scaffold. This treatment increased the formation of blood vessels and the synthesis of bone in rats with a critical-sized femoral lesion.

In addition to BMPs and VEGF, several other growth factors and signaling molecules have been investigated for their potential in promoting bone regeneration, including:

### 6.1 Fibroblast growth factors (FGFs)

Fibroblast growth factors, including FGF-2 and FGF-18, have been shown to induce angiogenesis, increase the growth of osteoblasts, and improve the process of MSCs transforming into bone cells (95).

### 6.2 Platelet-derived growth factors (PDGFs)

Platelet-derived growth factors, namely PDGF-A, have a vital function in the process of bone healing. They stimulate the growth and movement of chondrocytes, while also preventing cell death (96). According to Shah et al. (97), they enhance bone regeneration by stimulating cell proliferation, increasing the growth of new blood vessels, and activating macrophages. This is accomplished via increasing the expression of GIT1 and phosphorylation of Rac1, which then triggers the activation of the ERK1/2 pathway (98).

### 6.3 Wingless-type (Wnt) signaling molecules

The Wnt signaling pathway is a fundamental controller of osteoblast development and bone production. Its primary components, such as Wnt ligand, β-catenin, and transcriptional factors RUNX2, play vital roles in this process (99). Wnt-mediated signals have a significant role in the process of bone resorption, as shown by the research conducted by Maeda et al. (100). The Wnt/β-catenin canonical pathway plays a crucial role in regulating the process of osteogenetic differentiation, osteoblast proliferation, and the creation of bone matrix. WNT3A and WNT co-receptors LRP5 and LRP6, which are specific components of the Wnt pathway, have been identified as having significant influence on osteoblasts.

### 6.4 Transforming growth factor-beta (TGF-β)

**FIGURE 5:**
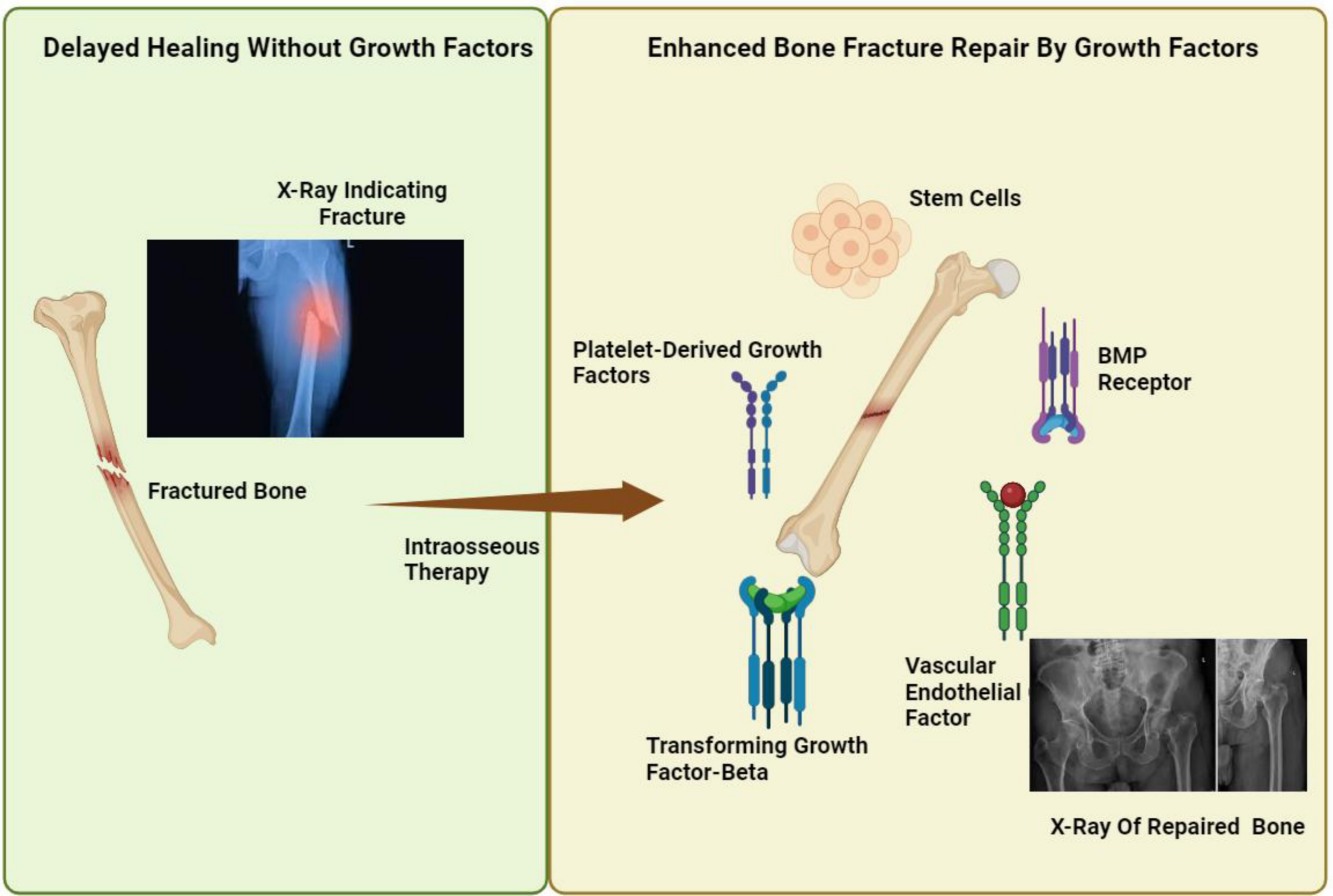
Growth factor-mediated enhancement of bone fracture healing through stem cell activation and signaling pathways, figure contrasts delayed bone healing without growth factors with enhanced repair through intraosseous therapy, highlighting the efficacy of platelet-derived growth factors, BMP Receptors, and Transforming Growth Factor-Beta in conjunction with stem cells, as evidenced by pre- and post-treatment X-rays.

Transforming growth factor-beta has been shown to control several facets of bone remodeling, such as the activity of osteoblasts and osteoclasts, as well as the differentiation of MSCs (101). It is secreted during the process of bone resorption by osteoclasts, attracting mesenchymal stem cells to the areas where bone is being broken down and stimulating the formation of osteoblasts (102). TGF-β inhibits the formation of osteoclasts generated by RANKL by reducing the expression of NFATc1 (103) (Figure 5).

## 7 Clinical applications of intraosseous therapy

Multiple studies have shown that intraosseous treatments have the ability to enhance bone fracture healing and address non-unions. Hernigou et al. (104) found that 88% of atrophic non-unions treated with autologous bone marrow concentrate (BMC) achieved union at an average period of 4.5 months. The study conducted by Calori et al. (105) revealed that the combination of mesenchymal stem cell (MSC) treatment with a scaffold led to a considerably greater percentage of bone union (86.7%) compared to using a scaffold alone (63.6%) in cases of non-union. Giannoudis et al. (106) reported that 87.5% of non-unions treated with platelet-rich plasma (PRP) injections achieved union after 12 months. Benshabat et al. (107) found that in cases with clavicle fractured non-union, there was a 95.2% success rate in achieving union, with an average period of 4.5 months for the union to occur.

### 7.1 Spinal fusion

Intraosseous treatments have demonstrated favorable outcomes in augmenting the rates of spinal fusion and facilitating the formation of solid bone connections in spinal fusion surgeries. Multiple research projects have examined the utilization of stem cells obtained from bone marrow, platelet-rich plasma (PRP), and other treatments administered into the bone in the context of spinal fusion procedures. The study conducted by Holmes et al. (108) shown that adipose-derived stem cells (ASCs) produced similar fusion outcomes to bone marrow cells (BMCs) in a rat model. Chotivichit et al. (109) found that the fusion rates were greater when MSCs were added to allografts in human patients. Particularly, a significant difference in fusion rates after 12 and 24 months was observed. A randomized controlled experiment conducted by Kubota et al. (110) examined the use of platelet-rich plasma (PRP) in single-level instrumented posterolateral lumbar fusion. The trial had a total of 60 patients, with half of them (30 patients) undergoing the conventional fusion technique together with Platelet-Rich Plasma (PRP) treatment, while the other half received only the usual fusion procedure. During the 12-month follow-up, the PRP group had a markedly greater fusion rate (86.7%) in comparison to the control group (63.3%).

### 7.2 Craniofacial and maxillofacial reconstruction

Intraosseous treatments have demonstrated encouraging outcomes in craniofacial and maxillofacial reconstruction, namely in facilitating bone regeneration and restoration. Multiple research have investigated the utilization of stem cells, growth factors, and other regenerative products in this particular domain. Kaigler et al. (111) conducted a preliminary clinical investigation examining the use of stem cells obtained from bone marrow for the purpose of regenerating alveolar bone. The study included 10 patients who were administered stem cell-loaded scaffolds for the purpose of alveolar ridge augmentation. The findings demonstrated substantial enhancements in bone growth and implant stability when compared to the control group. Pagni et al. (112) emphasize the capacity of cell-based treatments, such as bone repair cells and stem cells, to improve the regenerative response for repairing wounds in the craniofacial region. These treatments have demonstrated efficacy in the restoration of bone and soft tissue abnormalities in the oral and craniofacial regions.

### 7.3 Osteonecrosis and avascular necrosis

Intraosseous treatments have demonstrated encouraging efficacy in the management of osteonecrosis and avascular necrosis. Multiple studies have examined the utilization of stem cells, platelet-rich plasma (PRP), and other regenerative treatments to enhance bone regeneration and revascularization in necrotic lesions. Sen et al. (113) documented favorable results when utilizing autologous bone marrow-derived mononuclear cells to treat osteonecrosis of the femoral head. This treatment led to notable enhancements in pain relief and joint functionality. The study conducted by Wang et al. (114) showed that bone marrow concentrate (BMC) therapy is highly efficient in treating early-stage osteonecrosis of the hip. The therapy achieved a success rate of 85% in repairing the lesions and preventing the condition from advancing to more severe stages. Raeissadat et al. (115) conducted a randomized controlled experiment and discovered that PRP injections had a substantial positive impact on clinical outcomes and resulted in a reduction in lesion size for patients with osteonecrosis of the femoral head, as compared to the control group.

### 7.4 Osteoporosis and bone defects

Intraosseous treatments have potential in treating osteoporosis and bone abnormalities by stimulating bone growth and repair. Multiple research have investigated the utilization of stem cells, growth factors, and other regenerative products in this particular setting.

Guan et al. (116) conducted a randomized controlled experiment to examine the efficacy of bone marrow-derived mesenchymal stem cells (BMSCs) in treating osteoporotic fractures. The study comprised 64 patients with osteoporotic fractures, and the group treated with bone marrow-derived mesenchymal stem cells (BMSC) had considerably higher rates of bone union and better development of callus compared to the control group. The study conducted by Saito et al. (117) showed that the treatment of iPSC-MSCs resulted in a substantial enhancement in bone mineral density, trabecular bone volume, and bone strength when compared to the control group. In a study done by Sierra-García et al. (118), a systematic review and meta-analysis of 28 research revealed that bone morphogenetic proteins (BMPs) have a substantial positive effect on bone regeneration and defect repair when compared to control groups.

## 8 Critical evaluation of evidence and safety considerations

Despite the compelling molecular rationale and positive preclinical findings, the clinical translation of intraosseous therapy is still varied and, in many cases, based on inadequate evidence. A significant percentage of the literature is based on *in vitro* research, small-animal models, or uncontrolled clinical case series, which may overstate therapeutic efficacy while failing to capture clinically relevant adverse effects.

Stem cell-based intraosseous therapies, particularly those utilizing mesenchymal stem cells (MSCs), have shown strong osteogenic and angiogenic effects in preclinical settings. However, clinical outcomes have been more diverse, with patient age, comorbidities, cell source, concentration, and administration technique all having an impact. Furthermore, most clinical studies are constrained by small sample sizes, short follow-up durations, and absence of proper control groups, which reduces confidence in long-term efficacy.

Similarly, platelet-rich plasma (PRP) therapies vary significantly due to changes in preparation techniques, platelet concentrations, activation modalities, and injection strategies. This unpredictability has led to inconsistencies in clinical outcomes and restricts the comparability of published studies. While some randomized trials and meta-analyses demonstrate benefits in specific indications, others show neutral results, emphasizing the importance of standardized techniques and indication-specific evaluation. Such as the use of growth factors, such as recombinant human bone morphogenetic protein-2 (rhBMP-2), demonstrates the significance of essential safety evaluation. Although rhBMP-2 has been shown to have strong osteoinductive properties and improve fusion or healing rates in certain contexts, its clinical use has been linked to adverse events such as ectopic or heterotopic ossification, inflammatory reactions, osteolysis, and, in some cases, neurovascular complications. These safety issues have sparked ongoing debate about proper dose, indications, and risk-benefit balance (119).

The level of evidence varies greatly among clinical indications, including non-union fracture, spinal fusion, osteonecrosis, and osteoporosis. Some applications are backed by randomized controlled trials or systematic reviews, whilst others depend mostly on early-stage or observational evidence. As a result, intraosseous therapies should be seen as complementary or exploratory techniques in many therapeutic contexts, rather than generally accepted standards of care. Future advancements will necessitate well-designed, sufficiently powered clinical studies with standardized outcome measures, long-term safety monitoring, and open reporting of both positive and negative findings. Such efforts are critical for establishing acceptable patient selection criteria, optimizing therapy procedures, and ensuring safe and evidence-based clinical adoption.

The increasing evidence in favor of intraosseous therapy is indicative of both rapid methodological advancement and significant biological promise. However, because the topic is still undergoing active translation, interpretation across studies needs to be contextualized. While methodological variation in cell sources, preparation protocols, distribution strategies, biomaterials, and outcome measurements currently restrict direct cross-study comparability, positive outcomes are more often reported, a common pattern in new regenerative technologies. Many clinical trials prioritize feasibility and biological response, resulting in small sample sizes, variable blinding, and reliance on surrogate endpoints, whereas standardized hard outcomes, such as validated radiographic fusion, reoperation rates, and objective functional measures, are less consistently reported. These characteristics do not decrease the therapeutic potential of intraosseous therapies; rather, they characterize the field’s current maturity and identify clear goals for the design of next-generation, conclusive clinical trials.

## 9 Challenges and future perspectives

Although intraosseous therapies use autologous cells and biocompatible materials, immune responses continue to be a crucial driver of therapeutic outcome. Following implantation, biomaterials and transplanted cells interact with the host immune system, specifically macrophages, which play an important role in bone repair. Classically activated M1 macrophages cause pro-inflammatory responses that can hinder tissue integration, whereas alternatively activated M2 macrophages promote angiogenesis, matrix remodeling, and bone formation. An imbalance that favors extended M1 polarization could lead to fibrosis, poor scaffold integration, or decreased stem cell efficacy. In order to boost regenerative results, new strategies seek to develop immunomodulatory biomaterials and delivery methods that facilitate a prompt M1-to-M2 transition.

The biological potency of mesenchymal stem cell therapies varies greatly and is controlled by donor and manufacturing parameters. Donor age, health state, and comorbidities like diabetes or osteoporosis have a considerable impact on cell yield, proliferative capacity, and osteogenic differentiation potential. Furthermore, *in vitro* growth procedures, passage number, and culture conditions can influence cellular phenotypic and functionality. This variability affects standardization, repeatability, and cross-study comparison, posing a significant hurdle to consistent therapeutic efficacy. To address these issues, new donor selection criteria, standardized production processes, and reliable potency testing would be required.

Moreover, Biomaterial implantation invariably results in a foreign body response characterized by protein adsorption, immune cell recruitment, and fibrous encapsulation, which can jeopardize scaffold integration and functionality. Furthermore, mismatches between biomaterial degradation rates and bone regeneration rates might result in premature loss of mechanical support or protracted persistence, which impedes tissue remodeling. In load-bearing applications, stress shielding from stiff metallic implants might further decrease physiological load transmission, resulting in bone resorption and poor long-term outcomes. These problems underscore the importance of biomaterials with variable degradation patterns, suitable mechanical qualities, and immunologically friendly surface features.

Beyond biological concerns, economic and regulatory issues have a significant impact on the clinical translation of intraosseous medicines. Cell-based and sophisticated biomaterial therapies frequently incur expensive manufacturing, quality control, and logistical expenses, restricting accessibility and uptake. Translational pathways are further shaped by regulatory frameworks. For instance, according to U.S. Food and Drug Administration guidelines, minimally manipulated autologous cell products encounter fewer regulatory obstacles than more-than-minimally manipulated or expanded cell therapies, which must go through rigorous approval procedures. One of the field’s biggest challenges is navigating these regulatory differences while proving cost-effectiveness and clinical value.

## 10 Conclusion

Intraosseous treatments have become a potential method for stimulating tissue regeneration and repair, specifically in the discipline of bone biology. This study emphasized the fundamental concepts and many methodologies utilized in intraosseous therapy, encompassing the application of bone marrow aspirate concentrates, platelet-rich plasma, stem cell-based therapies, biomaterials and scaffolds, and growth factors. These novel treatments have shown considerable promise in several medical contexts, including the mending of bone fractures and non-unions, the fusing of spinal vertebrae, the restoration of craniofacial and maxillofacial structures, the treatment of osteonecrosis and avascular necrosis, and the management of osteoporosis and bone abnormalities. Although intraosseous therapies show great potential, various obstacles need to be overcome. These include addressing regulatory and ethical concerns, ensuring standardization and quality control, facilitating clinical application and commercial feasibility, and conducting further research in areas such as personalized and precision medicine, advanced biomaterials and delivery systems, combination therapies, and *in vivo* monitoring and tracking. However, the ongoing investigation and advancement of intraosseous treatments have the potential to completely transform the area of tissue regeneration and repair. This might lead to better results and an improved quality of life for patients with different musculoskeletal diseases.
